# Quadriparesis and Broad Complex Tachycardia Secondary to Severe Hypokalaemia Induced by Distal Renal Tubular Acidosis as the Initial Manifestation of Sjogren’s Syndrome

**DOI:** 10.7759/cureus.38984

**Published:** 2023-05-13

**Authors:** Nidhi Kaeley, Anand M Gangdev, Soumya Subhra Datta, Utkarsh P Singh

**Affiliations:** 1 Emergency Medicine, All India Institute of Medical Sciences, Rishikesh, Rishikesh, IND; 2 Emergency Medicine, AII India Institute of Medical Science, Rishikesh, Rishikesh, IND

**Keywords:** distal renal tubular acidosis, sjögren's syndrome, ventricular tachycardia, hypokalaemia paralysis, hypokalaemia

## Abstract

Sjogren's syndrome is an autoimmune disorder characterized by lymphocytic infiltration of exocrine glands that typically manifests as dysfunction of the lacrimal or salivary glands. About one-third of Sjogren's syndrome patients exhibit systemic symptoms. In one-third of Sjogren's syndrome cases, renal tubular acidosis (RTA) is present. Hypokalemia is the most prevalent electrolyte disorder in patients with distal RTA. A middle-aged female presented to the emergency department with a complaint of sudden-onset quadriparesis followed by shortness of breath. Her arterial blood gas analysis revealed severe hypokalaemia and metabolic acidosis. ECG revealed broad complex tachycardia, which resolved after starting potassium infusion. On evaluating the cause of normal anion gap metabolic acidosis and hypokalaemia, she was found to have distal renal tubular acidosis (RTA). Furthermore, on evaluating the cause of distal RTA, her SSA/Anti Ro and SSB/Anti La levels came out to be elevated, and a probable diagnosis of Sjogren’s syndrome was made. Severe hypokalaemia leading to hypokalaemia quadriparesis and broad complex tachycardia as the initial manifestation of distal RTA due to Sjogren's syndrome is uncommon. Timely recognition and prompt replacement of potassium are key to improved outcomes. It is also important to note that Sjogren's syndrome should be taken into account even in the absence of sicca symptoms, like in our case.

## Introduction

Hypokalaemia may be caused by numerous conditions; however, severe hypokalaemia causing hypokalaemic paralysis is generally caused by type 1 renal tubular acidosis (RTA), primary hypokalaemic periodic paralysis, Gitelman syndrome, channelopathies, primary hyperaldosteronism, and thyrotoxic periodic paralysis [1]. Hypokalaemia can be seen in a third of patients with RTA and is mostly asymptomatic; however, if left untreated, it may lead to severe hypokalaemia [2]. Rao et al. reported that out of 31 patients with hypokalaemic paralysis, 13 had RTA (ten had proximal RTA and three had distal RTA); furthermore, out of these patients, three had Sjogren syndrome [3]. Hypokalaemia-induced paralysis is often described in the literature; however, severe hypokalaemia due to renal tubular acidosis in a patient with no clinical signs and symptoms of Sjogren’s syndrome is uncommon [4,5]. We report a case of a 50-year-old female who presented with severe hypokalaemia due to distal renal tubular acidosis associated with an underlying clinically inapparent Sjogren’s syndrome.

## Case presentation

A 50-year-old female was brought to the emergency department (ED) with a complaint of sudden onset quadriparesis for one day with intact sensorium and shortness of breath for five to six hours. There was a preceding history of vomiting for one week prior to her presentation to the ED. There was no history of fever, abdominal pain, loose stools, chest pain, palpitations, or decreased urine output. There was no history of vaccination, animal bite, trauma, or toxic substance ingestion. She had no history of dryness of the mouth or eyes, joint pain, or skin lesions. Also, there was no past history of any similar episode of quadriparesis.

**Figure 1:**
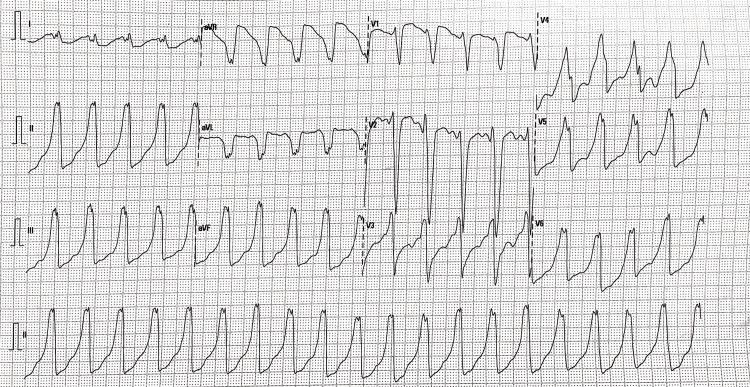
ECG suggestive of broad complex tachycardia

**Figure 2:**
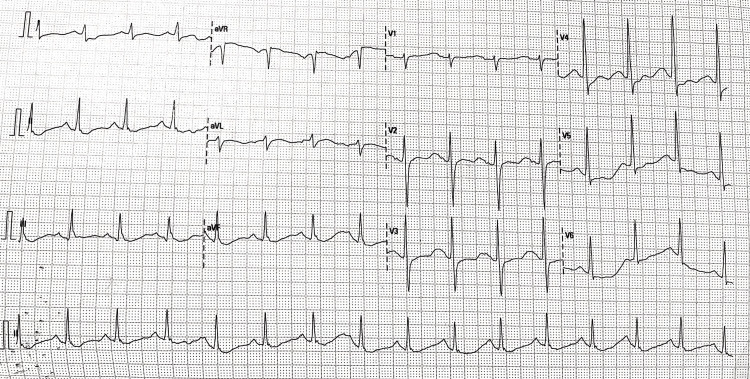
ECG showing normal sinus rhythm post-potassium replacement

On a primary survey, her airway was patent; she had a pulse rate of 102 beats per minute, a respiratory rate of 24 per minute, a blood pressure of 128/82 mm of Hg on her right arm in the supine position, and an oxygen saturation of 95% on five liters of oxygen via a face mask. The nervous system examination showed bilateral flaccid quadriparesis with a power of 2/5 on all limbs and equal proximal and distal weakness. Deep tendon reflexes were diminished bilaterally. The plantar reflex was muted on both limbs. There were no signs of sensory or cranial nerve involvement. Arterial blood gas analysis was suggestive of respiratory and metabolic acidosis (pH 7.13, PaCO2 44.1 mm of Hg, HCO3 14.1 mEq/L), severe hypokalaemia (K+ 1.09 mEq/L), hyperchloremia (115 mEq/L), and an anion gap of 14. The serum creatinine level was 1.6 mg/dL, and the blood urea nitrogen (BUN) level was 22 mg/dL. The serum albumin level was 3.5 g/dL. Her electrocardiogram was suggestive of broad complex tachycardia (Figure 1). The patient was intubated in view of severe respiratory distress and started on mechanical ventilation. A central line was placed in the right internal jugular vein, and hypokalaemia correction was started with an intravenous infusion of potassium chloride at a rate of 40 mEq/hr with continuous cardiac monitoring. The broad complex tachycardia resolved, and the rhythm reverted to normal sinus rhythm (Figure 2) two hours after the initiation of hypokalaemia correction. With further correction of hypokalaemia, quadriparesis resolved, and the patient was extubated on day three.

Urinary electrolytes showed a urinary sodium of 62 mEq/L, a urinary potassium of 18 mEq/L, and a urinary chloride of 66 mEq/L. The urinary anion gap was +14. The urine pH was 5.8. A positive urinary anion gap and high urinary pH were suggestive of impaired distal renal acidification. CSF analysis, non-contrast computerized tomography (NCCT) head, MRI brain, and whole spine were normal. High-resolution computed tomography (HRCT) of the chest was normal, suggesting no pulmonary involvement.

To look for the reason for RTA, antinuclear antibody (ANA) testing was done, which came out to be positive (ANA titre of 1:80) with a fine granular pattern. Anti-Ro/SS-A with a level of 84 (normal 20 U/ml) and anti-La/SS-B with a level of 98 (normal 20 U/ml) were found to be positive. Anti-histone, anti-Smith, anti-ribonucleoprotein, and anti-mitochondrial M2 antibodies were negative. Thyroid function tests (T3, T4, and TSH) were within the normal range. All of these findings pointed towards a probable diagnosis of primary Sjogren syndrome (SS). Schirmer’s test came out to be negative. The patient was not willing to undergo a salivary gland biopsy.

## Discussion

Primary Sjogren’s syndrome (SS) is an autoimmune disease that may involve both glandular and extra-glandular organs. Generally, glandular organs are involved before extra-glandular organs; however, sometimes the latter may be involved first. Therefore, in patients who present with involvement of extra-glandular organs first, it is challenging to consider the diagnosis of SS. Auto-antibodies against SS-A/Ro and anti-SS-B/La are specific for SS, and they become detectable much before the appearance of clinical symptoms [6,7]. Anti-Ro/SSA and anti-La/SSB antibodies are found in 50 to 70% of patients with primary Sjogren's syndrome [8].

In SS, renal involvement can be of two types: peri-epithelial (caused by lymphocytic infiltration) and extra-epithelial (caused by the deposition of immune complexes). Peri-epithelial lesions involve proximal tubules, intercalated cells, or the loop of Henle. Distal RTA is caused by the involvement of the intercalated cells and is seen in about one-fourth of the patients with SS [9].

Paralysis has been described as a presenting feature of distal renal tubular acidosis in relation to Hashimoto's thyroiditis, Sjogren's syndrome, and systemic lupus erythematosus [10,11]. Bulbar, ocular, and respiratory involvement are rare in hypokalaemic paralysis, but they may occur. Hypokalaemia in SS has been shown to have a greater risk of respiratory paralysis, but it is rarely the initial presentation [9].

Severe hypokalaemia can lead to life-threatening conditions such as ventricular tachycardia and respiratory paralysis, as in our case, and hence should be corrected promptly via central venous access. Ventricular tachycardia secondary to severe hypokalaemia should be initially treated with potassium replacement; however, if the rhythm is not reverted, we may consider using amiodarone and magnesium sulfate. Hypokalaemic paralysis secondary to distal renal tubular acidosis should be thoroughly evaluated for underlying primary disease, and definitive treatment should be initiated to prevent any relapse. Potassium should be replenished before bicarbonate therapy; otherwise, the trans-cellular shift of potassium into the cells can lead to severe hypokalemia. Immunosuppressive agents are not generally required [12].

## Conclusions

Severe hypokalaemia is a life-threatening manifestation of distal RTA, which can show significant ECG changes and paralysis often involving respiratory and bulbar muscles. Immediate electrolyte replacement and rigorous monitoring are required. In some cases, Sjogren's syndrome may go undiagnosed and manifest with complications of associated renal tubular acidosis; as in our case, acute-onset paralysis with respiratory muscle involvement and broad complex tachycardia were present due to hypokalaemia. Urine pH, urinary electrolyte level, and urinary anion gap are key for diagnosing renal tubular acidosis. Hypokalaemic paralysis should be worked up for underlying primary diseases even in the absence of classic symptoms. Sjogren’s syndrome (SS) is an autoimmune disorder that can involve both glandular and extra-glandular organs. Generally, involvement of glandular organs occurs before extra glandular organs, however vice-versa can also occur.
